# Supplementary material for: A fault-intrusion-tolerant system and deadline-aware algorithm for scheduling scientific workflow in the cloud
Source: PeerJ Comput Sci. 2021 Nov 2;7:e747. doi: 10.7717/peerj-cs.747 (PMC8576554; doi:10.7717/peerj-cs.747)
Supplement: Supplemental Information 6 [file peerj-cs-07-747-s006.zip › Myproject1.1/docs/org/cloudbus/cloudsim/core/predicates/package-summary.html]

org.cloudbus.cloudsim.core.predicates


JavaScript is disabled on your browser.


- Overview
- Package
- Class
- Use
- Tree
- Deprecated
- Index
- Help

- Prev Package
- Next Package

- Frames
- No Frames

- All Classes

# Package org.cloudbus.cloudsim.core.predicates

- Class Summary

  | Class | Description |
  | --- | --- |
  |  |  |
  | --- | --- |
  | Predicate | Predicates are used to select events from the deferred queue. |
  | PredicateAny | A predicate which will match any event on the deferred event queue. |
  | PredicateFrom | A predicate which selects events from specific entities.  The idea of simulation predicates was copied from SimJava 2. |
  | PredicateNone | A predicate which will **not** match any event on the deferred event queue. |
  | PredicateNotFrom | A predicate which selects events that have not been sent by specific entities. |
  | PredicateNotType | A predicate to select events that don't match specific tags. |
  | PredicateType | A predicate to select events with specific tags. |

- Overview
- Package
- Class
- Use
- Tree
- Deprecated
- Index
- Help

- Prev Package
- Next Package

- Frames
- No Frames

- All Classes
